# The Role of a PMI-Prediction Model in Evaluating Forensic Entomology Experimental Design, the Importance of Covariates, and the Utility of Response Variables for Estimating Time Since Death

**DOI:** 10.3390/insects8020047

**Published:** 2017-05-01

**Authors:** Jeffrey Wells, Lynn LaMotte

**Affiliations:** 1 Department of Biological Sciences, Florida International University, Miami, FL 33199, USA; 2 School of Public Health, Louisiana State University Health Sciences Center, New Orleans, LA 70112, USA; llamot@lsuhsc.edu

**Keywords:** postmortem interval, inverse prediction, experimental design, statistical methodology, validation

## Abstract

The most common forensic entomological application is the estimation of some portion of the time since death, or postmortem interval (PMI). To our knowledge, a PMI estimate is almost never accompanied by an associated probability. Statistical methods are now available for calculating confidence limits for an insect-based prediction of PMI for both succession and development data. In addition to it now being possible to employ these approaches in validation experiments and casework, it is also now possible to use the criterion of prediction performance to guide training experiments, i.e., to modify carrion insect development or succession experiment design in ways likely to improve the performance of PMI predictions using the resulting data. In this paper, we provide examples, derived from our research program on calculating PMI estimate probabilities, of how training data experiment design can influence the performance of a statistical model for PMI prediction.

## 1. Introduction

Although “forensic entomology” could refer to many other activities, the phrase almost always denotes a death investigation, and in particular using insects to estimate when death occurred [1]. The two familiar entomological postmortem clocks are the development of an individual insect and the succession of species on a corpse [2], and both processes may potentially be influenced by many factors [3]. Depending on the circumstances, the investigator may interpret a prediction of insect age or time a corpse was available to insects as the actual postmortem interval (PMI) or a minimum PMI (PMI_min_) [4,5].

We think that this prediction should be mathematically explicit, and that in most cases the prediction should be a range rather than a single value. If estimating the range using statistical analysis, the typical method is to calculate a confidence set, a step that we think can satisfy the National Research Council’s recommendation that any forensic science conclusion include an objective statement of the uncertainty associated with that conclusion [6].

Being able to calculate a probability for a PMI estimate would obviously improve casework, and we think it would be difficult to validate a PMI-prediction method if the estimate does not include confidence limits. It would be an unreasonable validation standard to expect the prediction to be exactly correct. How close to the correct value is close enough to support validity? Confidence limits provide the answer to that question. The coverage proportion should be greater than the nominal value, e.g., if calculating 95% confidence intervals, then at least 95% of predictions should include the true value.

Furthermore, it appears to us that forensic entomology researchers give almost no consideration to how choice of experimental design might influence PMI-prediction performance based on the resulting data.

### Some Specialized Terminology

When describing the relationship between development or succession and elapsed time, we refer to the amount of time that has elapsed (usually the time since oviposition/larviposition, i.e., age, or the time the corpse has been available to carrion insects, i.e., the succession interval (S.I.) [5]) as a *condition*, any other factor that influences development or succession rate (e.g., temperature) as a *covariate*, and that aspect(s) of a specimen or insect community that changes with time (e.g., larval length or species present on a corpse) as a *response*. Scientists concerned with PMI estimation try to understand (e.g., model) the condition/response relationship so as to be able to infer condition from response. To do this, one conducts a *training experiment* (producing *training data*, TD), in which one records the response(s) corresponding to known values of conditions.

Probably the more an investigator understands the correct condition/response relationship to use for a given death investigation, the more accurate a prediction of PMI will be. This relationship has been the topic of a great deal of published research into the effect of factors such as temperature e.g., [7], habitat e.g., [8], drug concentration e.g., [9], or sex of the insect e.g., [10]. However, modeling that relationship, such as fitting a regression line to the data, does not by itself specify how to predict condition from response. Exactly how to predict age or S.I. has received relatively little attention in the literature.

The purpose of this paper, then, is to persuade a forensic entomologist to think carefully about how experimental data will be used to predict carrion insect age or S.I. before she or he designs an experiment meant to support casework. To do this, we will describe examples, referred to as lessons, drawn from our own research program on inverse prediction and related statistical methods for PMI estimation [11,12,13,14,15,16].

## 2. Lesson 1: Employ an Unbiased Sampling Technique for Generating Training Data

This is an elementary aspect of good design for many kinds of experiments [17], and we examined the implications for a carrion insect age prediction model [18]. We were motivated by the fact that some authors deliberately collected biased samples by targeting the largest larvae in a single-age cohort, and by the fact that authors who claimed to take a random sample did not describe any randomization method [18], without which they could not have sampled even approximately at random [19]. Given that taking a random sample would require first physically isolating each individual from a rearing container, we doubt that an author who had done this would fail to mention it in the description of experimental methods.

**Figure 1:**
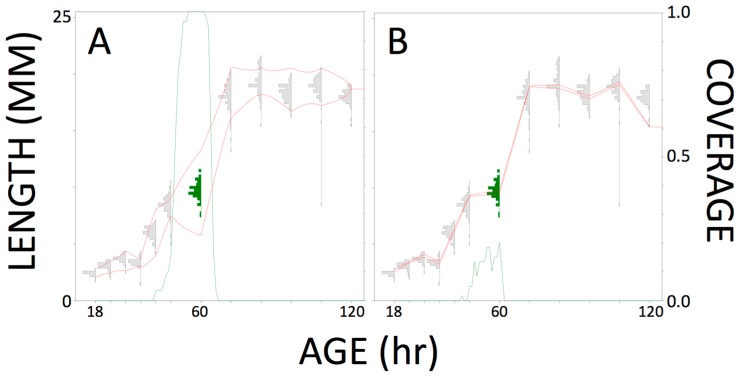
Blow fly growth data illustrating how inverse prediction model performance can depend on the design of the training data experiment. The histograms show the complete data set (*n* = 1405) of larval length as a function of age. The inverse prediction model (red lines defining a growth curve) reflect published sampling methods and were calculated based on subsamples of 10 from each cohort that were (**A**) randomly selected or (**B**) the largest individuals. Each green peak shows the coverage proportion for 95% prediction intervals on age from length for each larva in the 60 h cohort. Model A performed relatively well in that 100% of predictions included the true age and the line falls away steeply on each side. Model B performed poorly. From [18].

It was clear that sampling the largest larvae yielded an inaccurate prediction of age. For example, in Figure 1B, about 20% of the predictions included the true age. A model built from small random samples performed relatively well compared to using the much larger full data set, with 100% of predictions, shown by Figure 1A, including the true age. However, a random sample would require so much effort and would be so disruptive of development that the insects not selected would, we believe, not be suitable for inclusion in an older sample. Therefore, in most circumstances, one might as well sample (e.g., kill and measure) all insects in an age cohort. Perhaps a random sample, removing all insects in a rearing container without replacement at the chosen age followed by a randomization procedure to select a subset for data collection, might be worthwhile if the measurements were particularly time-consuming and/or expensive [18]. We think it is clear that the most commonly used sampling scheme in carrion insect development studies, i.e., repeatedly removing a small number of larvae from a rearing container, is a faulty experimental design and should be discontinued.

## 3. Lesson 2: Exceed the Minimum Sample Size for a Categorical Response

We proposed the only statistical method for predicting condition based on a categorical response [12], see also [20,21]. The original application was to estimate S.I. [5], but the same procedure can be used to predict insect age from stage of development.

Depending on the number of response categories and preferred level of statistical significance [12], there is a sample size, e.g., the number of training experiment carcasses observed for a given set of environmental conditions, below which it is not possible to ever reject a putative S.I. value (Table 1). In other words, below the smallest sample size, the training data do not provide enough statistical power for a prediction.

For example, the smallest sample size (e.g., the number of experimental pig carcasses or human corpses decomposed under a given set of circumstances) to possibly reject at the 5% level is 7, and at that the lowest amount of replication the method works for only two categories, e.g., the presence/absence of a single species during succession [12]. For two insect species (four response categories), the minimum sample size is 22, for three insect species it is 52, etc., and the analysis is likely to be more useful in practice if the sample size of the training data exceeds the smallest sample size [5].

The same logic applies to confidence limits on an estimate of insect specimen age from developmental stage [16]. Most published development data also included observations of one or more continuous responses, such as body length, but instar is a particularly reliable response because it is not affected by the mystery specimen preservation method. If the training data include six life stages (e.g., three larval instars, pupa, adult) then the smallest sample size is 37 insects per age [16].

We note that although sample size still plays a role in the performance of an age-prediction model based on continuous training data, there is no smallest sample size [14].

## 4. Lesson 3: The Practical Significance of a Covariate, or the Practical Value of a Response, Should Be Evaluated by Predictive Model Performance

Many authors examine the effect of one or more factors on carrion insect succession or development rate (see Introduction). For example, the discovery that food tissue type influenced larval growth rate led [22] to conclude “it is important to know where on a corpse larval material has come from”. The implication is that a predictive model based on training larvae reared on one substrate might be unacceptably inaccurate for predicting age of a mystery specimen that fed on a different substrate. The typical interpretation for an experiment such as this was to infer a practical effect from the discovery of a statistically significant effect e.g., [9,23,24].

**Figure 2:**
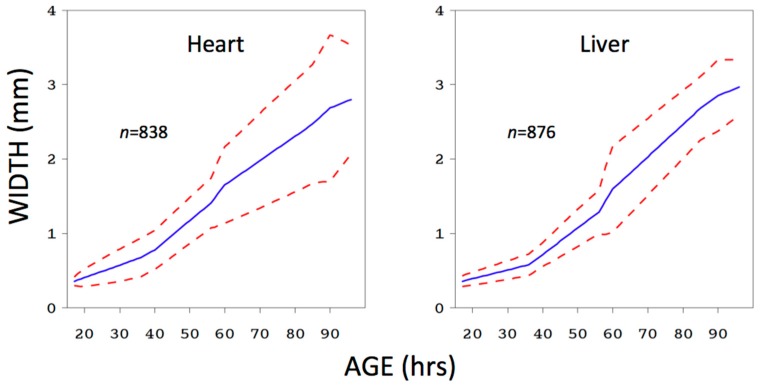
*Chrysomya megacephala* larval mean width as a function of age at 25.8 °C and fed either pork heart or pork liver. The data are from [25]. Red dotted lines show 95% inverse prediction confidence bands [12]. Although there was a significant effect of food type on size (*F* = 18.27, df = 8/700, *p* < 0.0001), when the liver model was used to predict the age of all heart larvae, more than 95% of inverse prediction confidence sets on age included the true age.

However, while we do not doubt that some covariates (temperature especially comes to mind) are of practical importance, so there should either be a match between training data and scene conditions, or some way to extrapolate between the two, a statistically significant effect does not automatically correspond to a practically important effect on prediction performance (Figure 2).

In this example, larval food type (pork heart vs. pork liver) had a highly significant effect on larval growth rate, but the model derived from liver was quite accurate when estimating the age of larvae grown on heart. Depending on the particular training data set and comparison, the outcome of these two approaches will not always conflict in this way, but given that a test of significance such as ANOVA does not answer the practical question of interest, while a measure of prediction performance such as the coverage rate does, we think that measuring prediction performance should be preferred.

Similarly, the fact that a particular variable changes with time since death does not by itself guarantee that its inclusion in the model will improve prediction performance. Table 2 shows a simple example of predicting larval age based on a multivariate response. In this case, a prediction of age based on length, width, and instar was no better than a prediction based on width and instar. The number of variables that potentially could be used to predict PMI is huge. One must decide what data to record and to not record during an experiment, and the effect on prediction performance may indicate whether or not to include that measurement in future studies. For example, insect sex influences development rate [26], but is it worthwhile, for example, to measure calliphorid larval sex when this requires relatively expensive equipment and/or reagents [27,28,29,30]? Note that we do not claim that including individual insect sex in the model is not worthwhile, just that this and other covariates need to be evaluated in this fashion.

For these reasons, we suggest that the practical importance of a covariate or the practical value of a response be assessed based on predictive model performance.

## 5. Conclusions

The most crucial practical application of forensic entomology is the prediction of carrion insect age or SI, which can then potentially be interpreted to support a forensic investigation such as when one concludes that the age of an insect equals PMI_min_. We argue that this prediction should be mathematically explicit, should yield a range of values rather than a single value, and that defining this range as a confidence set would conform to mainstream scientific practice.

To the extent that a reader agrees with our views, it follows that a central aim of forensic entomology research should be to optimize PMI prediction statistical model performance, something that can only be done if one employs such a statistical model. We hope that the examples presented here will better convey this message to readers who may have missed it within the mathematical language of our earlier publications.

## Figures and Tables

**Table 1:** Smallest sample size needed to reject a condition value at the 5% level based on a categorical response, such as when estimating succession interval from insect presence/absence or estimating insect age from life stage. For example, a succession pattern of a single species would have two response categories: present or absent on each training data (TD) experimental corpse at a given time since placement. If the number of TD corpses was 7–17, a time since death is rejected if zero TD corpses match the mystery specimen (MS, a corpse with a known set of insect species but unknown postmortem interval (PMI)). With 17–27 TD corpses, a time since death is rejected if zero or one TD corpse matches the MS, etc. Non-rejected values constitute a 95% confidence set on succession interval [12].

	Reject If ≤ No. TD Matching MS					
No. Response Categories	0	1	2	3	4	5
2	7	17	28	39	52	64
3	15	34	55	78	102	128
4	22	51	83	117	153	191
8	52	118	192	272	357	444

**Table 2:** 95% confidence limits about the age of a 56-hour-old *Chrysomya megacephala* larva using the multivariate method of [16]. Training data larvae were reared on pork heart at 25.8 °C and sampled at ages 17, 36, 40, 56, 60, 85, 90 and 96 h [25]. Age prediction models were based on combinations of the two continuous responses—body length (L) and body width (W)—and the categorical response, instar (I). An asterisk indicates rejection of an age (column values), so the prediction interval is shown by the gap in each row. The narrower the interval, the better the model performance.

MODEL	17	20	25	30	35	40	45	50	55	60	65	70	75	80	85	90
L	*	*	*	*	*	*	*						*	*	*	*
W	*	*	*	*	*	*	*							*	*	*
L, W	*	*	*	*	*	*	*							*	*	*
I	*	*	*	*	*					*	*	*	*	*	*	*
L, I	*	*	*	*	*	*				*	*	*	*	*	*	*
W, I	*	*	*	*	*	*	*			*	*	*	*	*	*	*
L, W, I	*	*	*	*	*	*	*			*	*	*	*	*	*	*

## Abbreviations

The following abbreviations are used in this manuscript:

IP	Inverse prediction
PMI	Postmortem interval
PMI_min_	Minimum postmortem interval
SI	Succession interval
TD	Training data
MS	Mystery specimen
